# Attitudes and Behaviors of Nurses and Nursing Students Toward Patients with Obesity: A Systematized Review

**DOI:** 10.3390/nursrep15020066

**Published:** 2025-02-12

**Authors:** Yaiza-María Arvelo-Rodríguez, Cristo-Manuel Marrero-González, Alfonso-Miguel García-Hernández

**Affiliations:** 1 Programa de Doctorado en Ciencias Médicas y Farmacéuticas, Desarrollo y Calidad de Vida, Universidad de La Laguna, 38200 Santa Cruz de Tenerife, Spain; 2 Facultad de Enfermería, Departamento de Enfermería, Universidad de La Laguna, 38200 Santa Cruz de Tenerife, Spain; cmarrerg@ull.edu.es (C.-M.M.-G.); almigar@ull.edu.es (A.-M.G.-H.)

**Keywords:** weight prejudice, nurse–patient relations, obesity, social stigma, stereotyping

## Abstract

**Background**: The prevalence of people with obesity is increasing worldwide, facing challenges in terms of discrimination and prejudice across all settings, including healthcare. **Objective**: The objective of this review is to compare and synthesize recent scientific literature regarding nurses’ behaviors and attitudes toward patients with obesity. **Methods**: A systematized methodology was employed, conducting a literature search of studies published in the bibliographic resources (Academic Search Complete, CINAHL Complete, Web of Science, and Scopus from 2018 to 2023); using specific terms combined with the Boolean operators, AND and OR. Inclusion criteria: Quantitative, qualitative, or mixed research conducted on nurses and/or nursing students in a context focused on the care of adult patients with obesity. Critical appraisal tools from the Joanna Briggs Institute (JBI) were used to evaluate the included studies, and their level of evidence was also determined. **Results**: Initially, the search yielded 166 articles, of which 14 were included in this review. The results found focused on the use of scales and tools heterogeneous to each other. The findings can be categorized into two main areas: studies investigating interventions aimed at reducing negative behaviors related to weight bias and prejudice, and studies focusing on assessing these attitudes. The evidence points in a consistent direction: nurses exhibit negative attitudes toward patients with obesity. **Conclusions**: The need for multilevel strategies, from clinical to academic training, to address this challenge is highlighted, alongside the development of research that complements the current evidence with a deeper and more detailed understanding of this phenomenon.

## 1. Introduction

Obesity is a multifactorial and complex trait disease resulting from the interaction between the current environment and human biology [1]. Its prevalence has been increasing in recent years, making it a public health issue. According to data from the World Health Organization (WHO) collected in 2022, one in eight people worldwide has obesity, with figures doubling among adults and quadrupling among adolescents since the 1990s [2].

There is robust literature on weight bias and discrimination against individuals with obesity within the healthcare system [3,4,5,6]. This discrimination manifests in restricted access to treatments and surgeries, communication barriers, and negative experiences that lead to avoidance of healthcare services, in addition to the physical, psychological, and emotional consequences of the stigma itself.

In 2020, 36 experts participated in an international statement aimed at ending obesity stigma in healthcare [7]. Among the thirteen recommendations were the recognition of obesity as a disease, increased awareness among healthcare professionals to identify stigmatizing situations, greater funding for obesity research, and the development of consistent policies to reduce weight-based inequalities.

Stigma is described as a characteristic that differentiates a person or group of people from others and, based on social beliefs, discredits or devalues those who bear it [8]. Regarding body size, weight stigma refers to the social devaluation and denigration experienced by individuals due to their body weight, resulting in stereotypes, prejudice, and discrimination [7].

Weight bias refers to judgments originating from beliefs generated by prejudices and stereotypes related to body size [9], fostering the development of negative attitudes. It is important to distinguish between implicit and explicit weight bias. Implicit bias operates automatically and without conscious intent, while explicit bias is characterized by consciously expressed attitudes [7].

Individuals, particularly those who have undergone nutritional treatments, regard the relationship established with healthcare professionals as a facilitating factor in adhering to recommendations [10]. However, weight bias among healthcare professionals negatively impacts the quality of healthcare. A higher body mass index (BMI) is associated with an increased likelihood of changing healthcare providers [11] and reduced utilization of healthcare services, as individuals avoid or delay seeking care [11,12]. These behaviors are rooted in stigmatizing experiences and poor-quality communication received from healthcare providers [11].

The identified gaps in the literature on weight stigma in healthcare settings are as follows:

- Effectiveness of educational interventions: Education is considered the most important recommendation for reducing weight-related stigma. While more research is needed, the growing interest in this issue is promising [6]. Strategies are designed for both current and future health professionals, though they are more commonly developed for students;
- Implications for clinical practice: Strategies for addressing obesity remain controversial. The traditional biomedical model, which focuses on weight loss, is often stigmatizing and contrasts with a more modern approach—Health at Every Size (HAES)—which prioritizes overall health without emphasizing weight [13]. As a social movement, HAES also generates tensions, such as the radicalization of the fat body, which can lead to the exclusion of individuals who are not considered “fat enough” [14];
- Cultural factors: It is essential to investigate how cultural differences influence professionals’ attitudes and the effectiveness of interventions. Regarding obesity, this condition extends beyond simply being an excess of fat; its experience, perception, and the expectations surrounding it are shaped by cultural values and interdependent social processes within a macrostructure where politics, economics, and government interact, shaping society’s interpretation of obesity [15];
- Stigma toward health professionals with obesity: Current literature primarily focuses on stigma toward patients. However, both health professionals and future practitioners also experience esthetic pressure regarding their bodies, as their physical appearance is socially perceived as a reflection of success. Additionally, they face the expectation of serving as role models for their clients [16].

The Code of Ethics and Conduct for European Nursing and Deontology states that nursing care is based on equity, inclusion, and appreciation of diversity, with the inherent professional obligation to respect the rights of patients and users, ensuring “*non-discrimination on grounds of age, color, creed, culture, ethnicity, disability or illness, gender, sexual orientation, nationality, political opinions, language, race, religious or spiritual beliefs, legal, economic, or social status*” [17] (p. 5). However, in 2006, Brown reported that nurses held negative attitudes toward adult patients with obesity, particularly in Western societies [18]. Therefore, considering the passage of time, cultural transformation, and the generation of current research on this subject, an updated synthesis is proposed. Exploring how nurses perceive their relationships with individuals with obesity promotes the understanding of this reality, contributing to the development of strategies to improve care quality and holistic, multidisciplinary, and patient-centered healthcare.

For these reasons, this review aims to shed light on the current behavior of nurses toward people with obesity. The goal is to encourage these professionals and related groups to reflect on and critically evaluate their daily practices to improve their approach toward this population.

The interaction between nurses and patients holds significant therapeutic potential that must be considered both in professional practice and in the training of future nurses [19]. Following Patricia Benner’s philosophy, this work considers nurses to include both qualified professionals and those in academic training, as nurses progress through various stages of professional development, from novices to experts [20]. In this context, nursing students are regarded as nurses in the process of developing their skills and competencies, gradually preparing for their professional roles.

## 2. Materials and Methods

A systematized literature review was conducted to compare and synthesize recent scientific literature on nurses’ behaviors and attitudes toward patients with obesity, with the aim of understanding and offering insights into this phenomenon [21]. The concept of a systematized review was first described by Grant and Booth [22] and is characterized by the inclusion of one or more methodological elements typical of systematic reviews.

To enhance the quality of this review, various elements from the guidelines of Preferred Reporting Items for Systematic Reviews and Meta-Analyses (PRISMA) [23] have been incorporated, specifically the PRISMA 2020 statement, were adopted.

This study follows a five-stage framework:

1. Identification of the research question/problem;
2. Identification of relevant studies through available literature;
3. Selection of studies through evaluation based on eligibility criteria;
4. Analysis of information and graphical representation of data;
5. Compilation, summary, and presentation of the findings.

These stages align with those described by authors such as Arksey and O’Malley [24] for scoping review methodologies and Whittemore and Knafl [25] for integrative reviews.

Initially, a preliminary search was conducted in PubMed and Google Scholar regarding weight discrimination in healthcare settings. This initial phase employed an iterative approach, identifying keywords and providing a general overview of the current state of the topic, primarily focusing on recent reviews on this subject.

During this pilot search, we decided to narrow the population exclusively to nurses and observed that a significant number of studies focused on nursing students. For this reason, we also included this population in our analysis.

Subsequently, the PCC strategy (population, concept, and context) [26] was used to define the problem:

- Population: nurses and nursing students;
- Concept: experiences, opinions, beliefs, and attitudes;
- Context: healthcare for patients with obesity.

Based on this, the following research question was formulated: What behaviors, attitudes, and beliefs are associated with nurses when providing care to people with obesity?

The search was conducted during August and September 2024 in the following bibliographic resources: Academic Search Complete (via EbscoHOST), CINAHL Complete (via EbscoHOST), PubMed (via the National Center for Biotechnology Information [NCBI]), Web of Science (WOS) (via WOS Complete), and Scopus (via Scopus-Elsevier). Medical Subject Headings [27] were used and grouped into three categories based on the strategy for defining the problem:

- Population: The terms identified included nurse, nursing staff, and nurse–patient relations;
- Concept: The terms identified included weight prejudice, bias, implicit, social stigma, and stereotyping;
- Context: The terms identified included obesity and overweight.

The described terms were then combined using the Boolean operators, AND and OR. To ensure the most up-to-date information, filters were applied to include studies published in the last five years, specifically between 2018 and 2023. The search strategy used for each bibliographic resource is detailed in Table 1.

Inclusion criteria included the following:

- Population characteristics: Studies focusing on nurses and nursing students, both male and female, were selected. Studies involving other healthcare professionals were included only if the majority of the results focused on nurses;
- Methodological design: Quantitative, qualitative, and mixed-method studies were selected;
- Concept: Studies addressing weight stigma in all its forms were included, whether focused on detecting it or on developing interventions and strategies to address it;
- Context: Studies in which the studied professionals provide healthcare to adult patients with obesity, both male and female, were selected. Articles published in English, Portuguese, and Spanish, without geographical limitations, were included.

Exclusion criteria included the following:

- Population characteristics: Studies focused on specialist nurses, particularly in obstetrics–gynecology, geriatrics, pediatrics, family and community care, and mental health. Studies involving children or adolescents were excluded;
- Methodological design: Gray literature, review articles, books, and editorials were excluded;
- Context: Studies in which healthcare professionals exclusively provide care to children or adolescents were excluded.

The results obtained were imported into the bibliographic reference manager Endnote^®^. Subsequently, the selection and screening of studies were conducted using the free version of the Rayyan^®^ tool. After removing duplicates, the records were assessed by title and abstract and organized into two groups: “include” and “exclude”. “include” records were retrieved for full-text screening.

Subsequently, a results table was used for data extraction and recorded in detail the following elements: authors, year of publication, country, research design, objectives, subjects or participants, instruments, and techniques used, main results, and scores obtained using the Joanna Briggs Institute (JBI) critical appraisal checklist, and JBI level of evidence [28]; these tools were employed to support the interpretation of the findings. Finally, a narrative synthesis of the results obtained was made. The literature was interpreted based on the results and conclusions of the selected studies.

JBI Critical Appraisal Tools for cross-sectional analytical studies [29], quasi-experimental studies [30], qualitative research [31], and randomized controlled trials [32] were used. These tools include a checklist with responses of “Yes”, “No”, “Unclear”, or “Not Applicable”.

## 3. Results

**Figure 1:**
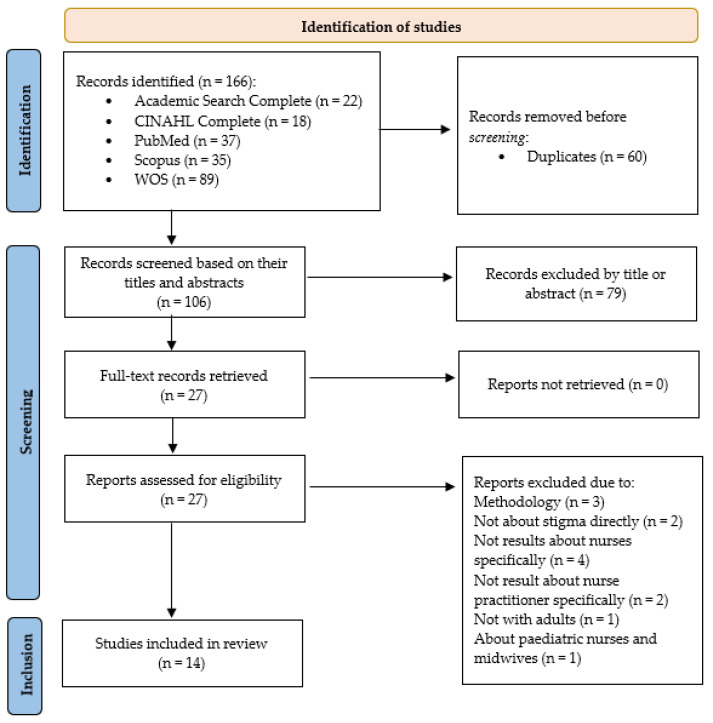
Article selection process following the PRISMA 2020 flow diagram.

The search strategy resulted in a total of 166 publications (Figure 1).

The highest number of results came from WOS (n = 89), followed by PubMed (n = 37), Scopus (n = 35), Academic Search Complete (n = 22), and CINAHL Complete (n = 18). A total of 60 duplicate articles were removed. An initial screening based on titles and abstracts was conducted (n = 106), excluding 79 studies. A full-text review of 27 publications was performed, resulting in the exclusion of 13 documents.

A total of 14 studies were selected for this review. The main characteristics extracted from the selected articles are presented in Table 2.

### 3.1. General Aspects of the Included Studies

Most of the studies were conducted in the United States [33,35,37,40,47,49,50,60], followed by Turkey [51,62]. Only one study was identified in New Zealand [39], Norway [53], Spain [57], and Germany [58]. The majority of the studies are quantitative in nature [33,35,37,40,49,51,53,57,58,60,62], one study employs mixed methods [47], and two studies use qualitative research [39,50]. Regarding the research objectives, the studies can be categorized into two groups: those that determine or explore the effectiveness of an intervention or educational program [33,37,39,40,47,49] and those aimed at measuring levels of prejudice or attitudes using scale-based instruments [35,51,53,57,58,60,62] and qualitative techniques [50].

Three of the fourteen included studies focus on licensed nurses [39,53,58]. Most of the research involves nursing students [33,35,37,40,47,49,50,51,57,60], although one study includes graduate students [35] and one examines both nurses and nursing students [62]. Some studies also include other professions, such as graduate students [35] and professionals [39].

#### Instruments and Techniques Used

Barra et al. [33] employed a proprietary scale called the Attitudes Toward Obese Persons Scale (ATOPS) [34] applied during pre- and post-educational intervention phases, with its validity established by an expert panel. Darling et al. [35] and Oliver et al. [49] utilized the Attitudes Toward Obese Persons Scale (ATOPS) and the Beliefs About Persons Scale (BAOP) [36] in their studies. Additionally, the ATOP scale was used exclusively by Joseph et al. [40], while the BAOP was used by Yilmaz et al. [62] and Llewellyn et al. [47].

Gajewski et al. [37] were the only ones to use the Jefferson Scale of Empathy–Health Professions Students (JSE-HPS), a version of the Jefferson Scale of Empathy [63] with minor modifications to suit the study population, as well as the Jefferson Scale of Patient Perceptions of Nurse Empathy (JSPPNE) [38] for the simulated patient conducting the role-play.

Joseph et al. [40] uniquely utilized the thinness-related subscale of the Sociocultural Attitudes Towards Appearance Questionnaire-4 (SATAQ-4) along with the Modified Differential Emotions Scale (mDES) [43] to measure positive emotions. Cognitive flexibility was assessed using the Cognitive Flexibility Inventory (CFI) [44]; self-compassion was measured with the Self-Compassion Scale—Short Form (SCS-SF) [45], and compassionate care was evaluated using the Compassion Competence Scale (CCS) [46]. This study also employed the Implicit Association Test (IAT) [42] to measure the strength of associations between concepts and stereotypes, aligning with the approaches of Robstad et al. [53] and Tracy et al. [60].

Explicit attitudes were measured by Tracy et al. [60] using a modified questionnaire for assessing cultural competence and communication [61], whereas Robstad et al. [53] used a scale based on specific stereotypes assessed in the IAT to rate feelings on a seven-point differential scale [54]. Additionally, these authors evaluated behavioral intention through four vignettes, where participants rated the likelihood of the scenarios occurring in real life on a seven-point semantic scale [56]. Similarly, Tannaberger et al. [58] employed a custom-designed questionnaire using a Likert scale to collect data on the frequency of providing care to individuals with obesity, the quality and availability of resources used, and perceptions of differential treatment compared to individuals with acceptable weight. They were the only study to use the Weight Control/Blame (WCB) subscale of the Antifat Attitudes Test (AFAT) [59]. The Anti-Fat Attitude (AFA) questionnaire [55] was used by Robstad et al. [53] and Rodríguez-Gázquez et al. [57]. Meanwhile, Fat Phobia Scale (FPS) [48] was utilized by Yilmaz et al. [62] and Llewellyn et al. [47].

Ozaydin et al. [51] uniquely employed the GAMS-27 Obesity Prejudice Scale [64] and the Stigma Scale [52].

In studies utilizing qualitative techniques, information was collected through semi-structured individual interviews in Hales et al. [39] and open-ended questionnaires in Hales et al. [39], Llewellyn et al. [47] and Oliver et al. [50] via reflective journals.

### 3.2. Main Findings

The main findings of the included studies, as well as the scores obtained using the Joanna Briggs Institute (JBI) critical appraisal checklist and the level of evidence according to established criteria, are presented in Table 3. No study was excluded based on the scores obtained from the checklist or the supported level of evidence.

Several studies show high levels of prejudice towards patients with obesity in students and nurses [51,62], identifying implicit and explicit biases [50,53,60]. Training programs have a positive impact on reducing prejudice [33,37,39,47,49], although a short exposure time may not be sufficient [40]. In addition, different factors influence attitudes towards people with obesity: females have higher levels of empathy and lower levels of prejudice [37,57] and personal contact with people with obesity [62] and older age [35] are associated with more positive attitudes. However, other studies find no gender differences [35,51].

Nursing students decrease their rejection as they progress through their academic training [47,57], although older students also demonstrate greater prejudice [51], possibly related to exposure to discriminatory and stereotypical situations in healthcare settings.

## 4. Discussions

This review includes studies published from 2018 onward. The findings can be categorized into two main areas: studies investigating interventions to reduce negative behaviors associated with weight bias and prejudice [33,37,39,40,47,49] and studies focused on evaluating these biases [35,51,53,57,58,60,62] among nurses and nursing students. The studies reviewed vary in objectives, methodologies, and the selection of subjects and participants. Intervention-based studies employ training, education, and simulation as tools to address this issue, while studies assessing negative attitudes primarily rely on quantitative scales and instruments.

Negative attitudes toward individuals with obesity are also confirmed in other contexts. In 2001, Puhl and Brownell [65] published the first review after several decades of research, revealing the stigma faced by individuals with obesity across various domains, including the workplace, healthcare, and education, highlighting the unjust treatment these individuals endure. Furthermore, false perceptions and beliefs about individuals with obesity promote discrimination and stigma through media, schools, and workplaces, including healthcare settings [1,66], as well as within families and society at large [66,67].

### 4.1. Overview of Nurses’ Attitudes and Behaviors

Most interventionist studies show that negative attitudes predominate before an intervention and improve afterward [33,37,39,47]. Educational and training interventions lead to a reduction in biases and the adoption of a less weight-centered perspective, focusing instead on patients’ needs. This finding aligns with other studies: López-Lara et al. [68] conducted an educational intervention that framed obesity as a multifactorial condition, shifting the perception of individuals with obesity among future healthcare professionals. However, it appears that interventions addressing weight bias have a limited impact [69,70], albeit a positive one for explicit bias but not for implicit weight bias [70]. This discrepancy may be attributed to insufficient exposure time [40] and the intensity required to achieve significant changes [49] in some of the included studies.

Nurses are exposed to caring for all types of patients regardless of their body size. However, prior contact with individuals with obesity appears to positively influence the attitudes of these professionals [51,62]. This finding aligns with Teachman et al. [71], who suggested that such exposure could act as a buffering factor. On the other hand, studies focused on professionals specifically trained to treat obesity reveal significant implicit weight bias among them [54,71], although it may be lower compared to professionals with less exposure. In this regard, a study conducted among dietitians found biased attitudes, though the levels were lower compared to other professional groups studied, with no association between the professionals’ BMI and their level of bias [72].

Similarly, a review by Moore et al. [69] noted that most research focuses on students rather than professionals, a finding consistent with the results presented. Only three of the fourteen included studies were conducted with licensed nurses [39,53,58], although one study included graduate students [35] and one examined both nurses and nursing students [62].

Our results show that more advanced students exhibit more negative attitudes compared to those with less academic experience [51]. These negative effects persist even when comparing licensed nurses with nursing students [62]. Conversely, the opposite trend was observed in other studies, with more advanced students showing more positive attitudes [35,57]. In this context, the available literature suggests that empathy seems to diminish with routine practice, as greater work experience in the health sciences field is associated with a lower development of empathy [73]. Conversely, other authors argue that greater moral maturity, acquired over time, is linked to a higher development of empathy [74].

Most of the included interventions promote empathy and awareness [33,37,39,47] consistent with other reviews [69,70]. Education and training are key to addressing this issue. Bocquier et al. [75] found a relationship between the level of weight bias in another professional category and the frequency of updating their knowledge. Similarly, in Thuan et al. [76], only 42% of respondents reported feeling prepared to care for overweight patients. In the case of nurses, continuous training could be crucial for fostering behavioral changes in clinical practice, as it updates and enhances their professional competencies [77]. This training and development largely depend on institutions and the programs they implement. Additionally, the responsibility to foster inclusive environments also falls on these institutions, requiring not only cross-cutting awareness and training but also providing the necessary equipment and materials to ensure appropriate care.

Two of the included studies noted that caring for individuals with obesity imposes greater effort and workload due to a lack of adequate resources to meet their needs [49,58]. Huang et al. [78] reported that the healthcare system is not adequately equipped to provide nursing care for individuals with obesity. On one hand, there is a shortage of human resources, as nursing activities require more time and trained personnel with competencies to manage these patients. On the other, there is a lack of infrastructure and equipment with specific materials for bariatric patients. The need for a larger number of nursing professionals is primarily related to the mobilization of these patients and hygiene procedures, which under standard conditions are typically performed by two people [79]. Therefore, an approach is needed that addresses not only the individual responsibility of the professional but also systemic issues to advance toward fairer and more equitable care for patients with these characteristics.

### 4.2. Contradictions and Complexities of the Findings

In three of the selected studies, no statistically significant relationship was found between the BMI of professionals and their levels of prejudice [51,53,60]. Compared to other findings, it appears that having a higher BMI is associated with higher levels of negative attitudes [80]. Schwartz et al. [54] note that these associations are specific to certain types of biases or prejudice metrics and are further influenced by other variables, such as age and sex, which explains the contradictory findings. For instance, in two studies, no differences were found when results were stratified by gender [35,51]. However, another included study found that female participants exhibited higher levels of empathy compared to males [37], in addition to scoring higher in the belief that individuals with obesity have less willpower [53]. In contrast, female participants scored lower in this belief in another study [57]. Additionally, the belief in weight control among professionals predicts greater discrimination in the treatment of individuals with obesity in clinical settings [58], aligning with Usta et al. [81]. This underscores the need for interventions aimed at reducing the belief in weight controllability [69], although this belief seems less ingrained among nurses [80].

The false belief that weight is controllable is strongly associated with stigmatizing behaviors, both from those who stigmatize and those who experience stigma. From the theoretical framework of stigma [82], and through the lens of Attribution Theory [83], developed in the context of mental illnesses [84,85], stigma is a cognitive–emotional process in which the causes and control of a condition—such as having excess weight—are attributed to individual responsibility and personal capacity for change. Similarly, Albert Bandura’s Self-Efficacy Theory [86] highlights how belief in one’s efficacy influences persistence, effort, and behavior. In the case of individuals with obesity, self-efficacy relates to the ability to overcome lifestyle-related barriers and the challenges of implementing changes toward health goals [87]. This theory has been used as a framework for planning and evaluating educational interventions for individuals with obesity [88], demonstrating its relationship with improved control over healthy habits [89] and internalized weight bias [90], where individuals with obesity adopt societal beliefs and stigma and apply them to themselves. Healthcare professionals, particularly nurses, play a key role in designing and implementing interventions that promote positive health changes in individuals. Professional-led strategies have proven effective in improving patients’ health outcomes [91].

Given the complexity of obesity and its associated implications, it is striking that research on weight bias among professionals often aims to determine and evaluate this issue through measurable levels, despite being a highly complex phenomenon influenced by numerous factors. Addressing this interrelation comprehensively from a positivist paradigm proves challenging. Jiménez-Domínguez [92] emphasizes the construction of social meanings and symbols in the social world that generate shared intersubjective realities.

Most of the presented findings come from quantitative research: only one study employed mixed methods [47] and only two used qualitative research [39,50], one of which explored an intervention [39]. This highlights the need for future research to adopt a more in-depth and detailed exploration of this reality. As Anguera Aguilada [93] describes, achieving maximum objectivity in understanding this complex issue requires qualitative or alternative research methods, a term preferred by Otálvaro and Páramo Bernal [94].

The purpose of this study is to provide a comprehensive overview of weight stigma among nurses and nursing students. Interest in weight stigma has been increasing; however, public health efforts have generally failed to recognize it as a barrier to addressing obesity [7]. Recommendations suggest that weight stigma should be tackled through education, practice, and research within the healthcare field [6]. The included studies, which primarily focus on nursing students, support the need to integrate competencies related to addressing weight stigma into nursing education [33,35,37,40,47,49,50,51,57,60]. These interventions can be particularly effective, as students’ beliefs and attitudes toward obesity are shaped and transformed during their training [6].

However, based on our findings, we recommend that interventions be layered and implemented under a multilevel approach. Integrating curricular, clinical, and structural components may offer significant potential to reduce this bias [95].

Clinical environments play a crucial role in shaping the beliefs and attitudes of healthcare professionals [6], and nursing students undergo substantial practical training as part of their education. This justifies the application of a multilevel approach, as both current and future professionals are interconnected within a reciprocal exchange, where nursing students witness and internalize weight stigma as it is perpetuated in clinical settings [95].

At the clinical level, alternative models could be incorporated to manage patients with obesity through a less weight-centric approach, prioritizing individual well-being and overall health rather than reducing patients solely to their weight [13]. Additionally, the integration of appropriate equipment and resources tailored to the needs of these patients could influence nurses’ perceptions, particularly regarding the belief that caring for patients with obesity is burdensome [95] or challenging [6].

At the research level, recommendations emphasize the need for robust empirical evidence, particularly through longitudinal studies and randomized controlled trials (RCTs) involving large populations [6]. However, considering our findings and the complexity of weight stigma, it is essential to further emphasize the need to understand obesity as a highly complex social phenomenon, shaped by a multitude of factors and contexts that also warrant investigation.

Nursing is a profession dedicated to caring for people, heavily influenced by the humanist perspective. Nogales Espert [96] referred to the art of caregiving as an expression of empathy, encouraging nurses to cultivate their sensitivity in pursuit of both technical and emotional excellence in their practice. This review reveals current negative findings [50,51,53,58,60,62] in the form of attitudes, prejudices, and beliefs among nurses toward patients with obesity. Being a healthcare professional does not guarantee the absence of bias, as physicians, dietitians, psychologists, physiotherapists, occupational therapists, speech therapists, podiatrists, and professionals in physical activity and sports have all demonstrated explicit and/or implicit biases against people with obesity [4]. However, as noted by Sikorski et al. [80], nurses, compared to other professionals, tend to exhibit more positive attitudes. Nonetheless, the solution does not lie in finding solace in the challenges faced by others.

Ultimately, it is important to remember that people seeking care within the healthcare system expect to be treated with respect and empathy. Therefore, it is essential for the healthcare system to function as it should create a safe and judgment-free space where every person can receive the help they need without fear of discrimination.

### 4.3. Limitations of the Review

Given that knowledge on weight stigma among nurses is dispersed and homogeneous, this review adopts a narrative analysis and interpretation approach to serve as a starting point for future, more structured reviews and studies. This type of review allows for the synthesis and exploration of this research field using a flexible methodology, providing a broader perspective on the topic [22]. However, despite incorporating several elements of systematicity, its limitations should be considered when interpreting the results:

- Search strategy: This review included studies published within the last five years and only in three languages, which may have excluded important evidence;
- Study variety: The diversity of methods and measures used across the studies made it difficult to synthesize findings and limited the possibility of conducting a comparative analysis;
- Quality of evidence: Although the JBI critical appraisal tools were applied, the variable quality of the included studies may influence the strength of the conclusions;
- Potential publication bias: Studies with unexpected or non-significant results may have been less likely to be published, affecting the comprehensiveness of the review;
- Applicability of results: The relevance of the selected studies in other cultural settings may be limited, as most were conducted in Western contexts;
- Focus on students: The findings predominantly center on nursing students, which may not accurately reflect the attitudes of experienced nurses.

## 5. Conclusions

The evidence gathered from studies addressing nurses’ bias toward patients with obesity indicates that nurses exhibit negative attitudes, behaviors, and beliefs toward people with this condition. This evidence is primarily derived from studies conducted with nursing students, which is concerning as they are future expert nurses. These findings highlight the need to incorporate competencies into university curricula to address weight bias. Efforts among licensed nurses should focus on increasing awareness, developing soft skills such as empathy, and improving the provision of human and material resources by the healthcare system to meet the needs of these patients.

The stigma associated with weight in healthcare settings poses a barrier to public health [7]. The findings highlight the need for significant changes in the training and education of future nurses, as well as the need to reformulate the clinical approach to patients with obesity, favoring a multilevel approach.

The main limitations identified in this review include variability in methodological quality and publication bias. Additionally, the findings may be limited due to the selection of primarily Western studies and the predominance of nursing students in the sample. Lastly, the use of narrative synthesis may have influenced the interpretation of the results.

As implications for practice, we believe this review contributes to the development of strategies aimed at improving care quality and patient-centered healthcare. It is necessary to conduct further studies that delve deeper into the complexity of this phenomenon and the nurse–patient relationship in the context of obesity.

## Figures and Tables

**Table 1:** Search strategy used.

Bibliographic Resources	Search Strategy
Academic Search Complete	(obesity or body weight or overweight) AND (nurse or nursing staff or Nurse-Patient Relations) AND (weight prejudice or bias, implicit or social stigma or Stereotyping)
CINAHL Complete	(obesity or body weight or overweight) AND (nurse or nursing staff or Nurse-Patient Relations) AND (weight prejudice or bias, implicit or social stigma or Stereotyping)
PubMed	(obesity or body weight or overweight) AND (nurse or nursing staff or Nurse-Patient Relations) AND (weight prejudice or bias, implicit or social stigma or Stereotyping)
Web of Science	TITLE-ABS-KEY (obesity OR “body weight” OR overweight) AND TITLE-ABS-KEY (nurse OR “nursing staff” OR “Nurse-Patient Relations”) AND TITLE-ABS-KEY (“weight prejudice” OR “bias, implicit” OR “social stigma” OR stereotyping)
Scopus	((TS = (obesity or body weight or overweight)) AND TS = (nurse or nursing staff or Nurse-Patient Relations)) AND TS = ((weight prejudice or bias, implicit or social stigma or Stereotyping))

**Table 2:** Key data from the selected studies.

Author (Year). Country Design	Objectives	Subjects or Participants	Instruments and Techniques
Barra et al. (2018) [33]. United States. Quantitative.	To determine the effectiveness of an awareness program on obesity.	Nursing students (n = 103). Third- and fourth-year students.	A weekly educational intervention was conducted. Before and after the intervention, participants completed the Attitudes Toward Obese Persons Scale (ATOPS) [34] and a questionnaire specifically designed for this study.
Darling et al. (2019) [35]. United States. Quantitative.	To evaluate undergraduate and postgraduate students’ attitudes toward individuals with obesity and compare nursing students with those from other professions.	Undergraduate and postgraduate nursing students (n = 403), postgraduate education students (n = 35), and postgraduate social work students (n = 88).	The Attitudes Toward Obese Persons Scale (ATOPS) and the Beliefs About Persons Scale (BAOP) [36] were used.
Gajewski et al. (2023) [37]. United States. Quantitative.	To evaluate the effectiveness of educational activities focused on weight bias to promote empathy.	Nursing students (n = 2021). First-year students.	Weight bias learning activities were conducted. The Jefferson Scale of Empathy–Health Professions Students (JSE-HPS) was used pre- and post-intervention. A simulation scenario was employed, followed by the Jefferson Scale of Patient Perceptions of Nurse Empathy (JSPPNE) [38] completed by the individual participating in the simulation.
Hales et al. (2018) [39]. New Zealand. Qualitative.	To explore the impact of using a simulation suit on participants’ attitudes and perceptions.	Registered nurses (n = 6) and a registered physiotherapist (n = 1) who regularly treated individuals with obesity.	A semi-structured individual interview was conducted pre- and post-simulation with a suit designed to mimic the shape and size of a person with obesity. Additionally, a pre-simulation questionnaire with five open-ended questions about perceptions of the daily challenges faced by people with obesity was completed.
Joseph et al. (2023) [40]. United States. Quantitative.	To determine the effectiveness of Loving-Kindness Meditation (LKM) in reducing weight bias.	Nursing students (n = 189). Loving-Kindness Meditation (LKM) condition (n = 80). Control condition (n = 109).	Both groups participated in a meditation session. The intervention group practiced LKM, while the control group engaged in a mindfulness-based body scan meditation. Both sessions lasted 10 min and were guided through an audio recording. Before the intervention, the thinness-related subscale from the Sociocultural Attitudes Towards Appearance Questionnaire-4 (SATAQ-4) [41] was used. After the meditation sessions, the following variables were measured by the researchers: Weight bias: IAT [42] was used; Positive emotions: The Modified Differential Emotions Scale (mDES) [43] was employed; Cognitive flexibility: The Cognitive Flexibility Inventory (CFI) [44] was utilized; Self-compassion: The Self-Compassion Scale—Short Form (SCS-SF) [45] was applied; Compassionate care: The Compassion Competence Scale (CCS) [46] was administrated; Attitudes toward individuals with obesity: ATOP [36] acted as an instrument.
Llewellyn et al. (2023) [47]. United States. Mixed Methods.	To evaluate weight bias in nursing students pre- and post-intervention using a communication tool and a simulation test.	Nursing students. Participants in the pre-survey (n = 47) and post-survey (n = 73). First semester.	The instruments used included the Fat Phobia Scale (FPS) [48], the Beliefs About Persons Scale (BAOP) [36], and open-ended questions, administered before and after a didactic course introducing the LEARN model. This model is based on patient-centered communication, emphasizing the patient’s concerns.
Oliver et al. (2022) [49]. United States. Quantitative.	To evaluate the effectiveness of incorporating deeper modules into Weight Bias Reduction (WBR) programs that promote self-reflection and critical thinking to improve attitudes and beliefs toward individuals with obesity.	Nursing students (n = 99). Participants in the intervention group: (n = 46). Participants in the control group: (n = 53). Third-year students.	All participants completed the ATOP and BAOP [36] questionnaires before and after each intervention. Intervention group: Received a WBR program with more intensive content. This intervention included weight-based case studies. Control group: Received the standard program.
Oliver et al. (2021) [50]. United States. Qualitative.	To explore nursing students’ reflections on weight bias in healthcare settings.	Nursing students (n = 197). Third-year students.	A reflective journal was used, where students progressively answered five questions over 15 weeks.
Ozaydin et al. (2022) [51]. Turkey. Quantitative.	To determine levels of prejudice and stigma toward individuals with obesity.	Nursing students (n = 233). Second-, third-, and fourth-year students.	The GAMS-27 Obesity Prejudice Scale and the Stigma Scale [52] were used. Data were collected through an online link providing access to the described questionnaires.
Robstad et al. (2019) [53]. Norway. Quantitative.	To examine the explicit and implicit attitudes of intensive care unit (ICU) nurses toward patients with obesity and whether these attitudes are associated with their intentional behaviors.	Qualified ICU nurses (n = 159). Participants were registered nurses with 1.5 years of continuous training in ICU care or a 2-year master’s degree in intensive care.	An online questionnaire was used, incorporating the following scales: Implicit bias: IAT [42]; Explicit bias: A scale based on specific stereotypes assessed in the IAT to rate feelings on a seven-point differential scale [54]. The Anti-Fat Attitude (AFA) questionnaire [55] was also used; Behavioral intention: Four vignettes were presented, and participants rated the likelihood of the described scenarios occurring in real life on a seven-point semantic scale (from very likely to very unlikely) [56].
Rodríguez-Gázquez et al. (2020) [57]. Spain. Quantitative.	To analyze changes in negative attitudes toward obesity throughout academic training.	Nursing students (n = 578). First-, second-, third-, and fourth-year students.	The AFA questionnaire [55] was applied, and data were collected in person during courses with the highest attendance rates.
Tanneberger et al. (2018) [58]. Germany. Quantitative.	To determine the belief that patients with obesity receive different care compared to non-obese individuals and to evaluate whether beliefs about weight control influence clinical practice.	Nurses in an intensive care clinic (n = 73).	The Weight Control/Blame (WCB) subscale of the Antifat Attitudes Test (AFAT) [59] was used. Additional questions, rated similarly to the utilized subscale, were included to gather data on the frequency of providing care to people with obesity, the quality and availability of resources used in their care, and the perception of whether nurses or their colleagues treated individuals with obesity differently compared to those with acceptable weight. Participants were also given the option to provide free-text responses to elaborate on perceived discrimination toward these patients.
Tracy et al. (2019) [60]. United States. Quantitative.	To determine whether explicit attitudes align with implicit beliefs.	Nursing students (n = 69). First semester.	A modified pre-survey questionnaire was used to evaluate cultural competence and communication [61], asking respondents to declare their preferences for fat or thin individuals, followed by the IAT [42] to assess unconscious preference for thin people.
Yilmaz et al. (2019) [62]. Turkey. Quantitative.	To evaluate whether prejudice toward people with obesity exists.	Nursing students (n = 190) and licensed nurses (n = 189).	Two scales were used: the Fat Phobia Scale (FPS) [48] and the Beliefs About Obese Persons Scale (BAOP) [36].

**Table 3:** Main findings of the selected studies, scores obtained using the JBI critical appraisal checklist, and level of evidence.

Author (Year)	Main Results	JBI
Barra et al. (2018) [33].	The pre-intervention survey revealed that more than half of the students held negative opinions toward patients with obesity. A significant positive change in students’ biases was observed after the training sessions, with students expressing remorse upon recognizing that their weight biases impacted the quality of care provided.	In total, 6 out of 9 points on the critical appraisal checklist for quasi-experimental studies. Level of Evidence: II
Darling et al. (2019) [35].	No differences were found between male and female scores on either scale. However, older participants were associated with more positive beliefs and attitudes. Nursing students appeared to have fewer positive attitudes compared to students from other professions.	In total, 6 out of 8 points on the critical appraisal checklist for cross-sectional analytical studies. Level of Evidence: IV
Gajewski et al. (2023) [37].	Empathy levels were high both before and after the learning activities. In one cohort, significant changes in empathy levels were demonstrated before and after the intervention. Female participants scored higher in empathy compared to males.	In total, 6 out of 9 points on the critical appraisal checklist for quasi-experimental studies. Level of Evidence: II
Hales et al. (2018) [39].	Participants experienced the challenges faced by individuals with obesity, gaining a deeper understanding of how this condition affects physical challenges and social interactions, often resulting in social isolation. The use of simulation suits among healthcare professionals had a positive impact on reducing weight stigma.	In total, 9 out of 10 points on the critical appraisal checklist for qualitative research. Level of Evidence: III
Joseph et al. (2023) [40].	Participants in the intervention group experienced significantly higher levels of emotions such as gratitude and love. No significant differences were found in other variables, such as self-compassion, weight bias, cognitive flexibility, compassionate care, or positive attitudes toward individuals with obesity. These findings suggest that a 10-min exposure may not be sufficient to yield significant differences. Additionally, baseline data were not collected to avoid participant bias.	In total, 11 out of 13 points on the critical appraisal checklist for randomized controlled trials. Level of Evidence: I.
Llewellyn et al. (2023) [47].	Quantitative Results: Significant differences were observed, with respondents being less likely to agree that obesity results from a lack of love, overeating, or lack of physical exercise. Positive trends were also noted regarding qualities such as strength, self-control, and resilience. Qualitative Results: Students adopted a less weight-centered approach, focusing more on the individual and tailoring care to the patient’s needs.	In total, 6 out of 8 points on the critical appraisal checklist for cross-sectional analytical studies. 7 out of 8 points on the critical appraisal checklist for qualitative studies. Level of Evidence: III.
Oliver et al. (2021) [49].	BAOP scores, reflecting beliefs about individuals with obesity, improved significantly more in the intervention group than in the control group. However, no significant changes were observed in the ATOP scale results, which measure attitudes toward individuals with obesity.	In total, 11 out of 13 points on the critical appraisal checklist for randomized controlled trials. Level of Evidence: I.
Oliver et al. (2021) [50].	Observed Implicit and Explicit Weight Bias: Students reported that nurses made derogatory comments to patients with obesity. Weight Bias Due to External Factors: Students noted that caring for patients with obesity posed a greater burden compared to patients of acceptable weight, partly due to the lack of an accommodating environment for treating these individuals. These aspects created a contrast between what students learned about weight bias and their clinical practice experiences.	In total, 9 out of 10 points on the critical appraisal checklist for qualitative research. Level of Evidence: III.
Ozaydin et al. (2022) [51].	High levels of prejudice and stigmatization toward individuals with obesity were detected, with a positive correlation between stigmatization and prejudice levels. Fourth-year students demonstrated significantly higher prejudice levels than younger students. No differences were found for other variables such as gender, place of residence, BMI of the respondent, economic level, the presence of first-degree relatives with obesity, or interactions with individuals with obesity during clinical practice.	In total, 8 out of 8 points on the critical appraisal checklist for cross-sectional analytical studies. Level of Evidence: IV
Robstad et al. (2019) [53].	A greater preference for thin individuals was detected, both in implicit and explicit attitudes. No association was found between explicit and implicit attitudes and the self-reported weight of the study participants. However, male participants scored higher in the belief that individuals with obesity have less willpower. Explicit and implicit attitudes were not associated with behavioral intention.	In total, 6 out of 8 points on the critical appraisal checklist for cross-sectional analytical studies. Level of Evidence: IV.
Rodríguez-Gázquez et al. (2020) [57].	First-year students scored higher overall on the scale, indicating a stronger attitude against obesity. Female participants showed lower values in terms of aversion and perceived willpower. Attitudes became less negative as the academic years progressed.	In total, 6 out of 8 points on the critical appraisal checklist for cross-sectional analytical studies. Level of Evidence: IV.
Tanneberger et al. (2018) [58].	A significant association was found between healthcare professionals’ weight control beliefs and their perception that individuals with obesity were treated differently compared to those of acceptable weight, both by their colleagues and by the professionals themselves. Weight control beliefs were the only significant factor predicting the perception of discrimination by nurses toward patients with obesity. Nurses reported a lack of adequate resources, which increased the perception that care required greater intensity.	In total, 6 out of 8 points on the critical appraisal checklist for cross-sectional analytical studies. Level of Evidence: IV.
Tracy et al. (2019) [60].	More than 65% of respondents demonstrated a significant difference between their self-reported attitudes toward individuals with obesity and the results of the IAT on implicit attitudes. No relationship was found between the BMI of the respondent and their preference for body mass in others.	In total, 6 out of 8 points on the critical appraisal checklist for cross-sectional analytical studies. Level of Evidence: IV
Yilmaz et al. (2019) [62].	The majority of nursing students and licensed nurses reported negative attitudes and beliefs toward individuals with obesity. In both the FPS and BAOP results, the proportion of negative responses was significantly higher among licensed nurses compared to nursing students. Participants with obesity exhibited more positive attitudes, and having relatives with obesity was also associated with a more positive attitude.	In total, 7 out of 8 points on the critical appraisal checklist for cross-sectional analytical studies. Level of Evidence: IV.

## Data Availability

No applicable.

## Public Involvement Statement

There was no public involvement in any aspect of this research.

## Guidelines and Standards Statement

This manuscript was prepared following one or more methodological elements of the Preferred Reporting Items for Systematic Reviews and Meta-Analyses (PRISMA 2020 statement). The review protocol has been registered and is available at https://osf.io/btxu8/ (DOI 10.17605/OSF.IO/BTXU8) (accessed on 6 February 2025).
